# Increasing prevalence and incidence of domestic violence during the pregnancy and one and a half year postpartum, as well as risk factors: -a longitudinal cohort study in Southern Sweden

**DOI:** 10.1186/s12884-016-1122-6

**Published:** 2016-10-26

**Authors:** Hafrún Finnbogadóttir, Anna-Karin Dykes

**Affiliations:** 1 Department of Care Science, Faculty of Health and Society, Malmoe University, Malmoe, Sweden; 2 Department of Health Sciences, Medical Faculty, Lund University, Lund, Sweden

**Keywords:** Domestic violence, Longitudinally, Pregnancy, Postpartum, Prevalence, Incidence, Risk factors

## Abstract

**Background:**

Domestic violence is a global health problem as well as a violation against human rights. The *aim* of this study was to explore prevalence and incidence of domestic violence during pregnancy and 1 to 1.5 years postpartum as well as to explore the history of violence among new mothers in the southwestern region of Sweden. In addition, the aim was to explore the association between domestic violence postpartum and possible risk factors.

**Methods:**

This is a longitudinal cohort-study including pregnant women ≥ 18 years of age. Total 1939 pregnant women were recruited to the study and requested to answer three questionnaires (QI-III) during pregnancy and postpartum. Statistical analysis were descriptive statistics, logistic regression and multiple regression with Odds ratios (OR) and 95 % confidence intervals (95 % CI).

**Results:**

The response rate for those who received the Q-III (*n* = 755) at a Child Welfare Center was almost 97 % (*n* = 731). When all three questionnaires were answered the prevalence of domestic violence during pregnancy irrespective of type or severity was reported by 2.5 % (*n* = 40/1573). At 1 to 1.5 years postpartum the prevalence of domestic violence had increased to 3.3 % (*n* = 23/697). The incidence was 14 per 1000 women during pregnancy and 17.2 per 1000 women postpartum. The strongest risk factor for domestic violence reported at1-1.5 years postpartum was *a history of violence* whereby all of the women (*n* = 23) who had revealed their exposure to domestic violence postpartum also reported *a history of violence* (*p* < 0.001). Being *single/living apart* gave a 12.9 times higher risk for domestic violence postpartum (AOR 12.9; 95 % CI: 4.5–37.1). Having *several symptoms of depression* and a *low score on the SOC-scale* gave a 3.5 and 3.0 times higher risk respectively (AOR 3.5; 95 % CI: 1.2–10.4) and (AOR 3.0; 95 % CI 1.1–8.3).

**Conclusion:**

Domestic violence increases as the pregnancy develops and postpartum. A history of violence and being single/living apart may be strong indicators for domestic violence during pregnancy as well as postpartum. Also, having symptoms of depression are associated with domestic violence both during pregnancy and postpartum. Collaboration between health care providers at Antenatal and Welfare centres is essential.

## Background

Domestic violence (DV) is a complex global public health problem as well as a violation against human rights [1]. The definition of DV, as used in this study, is in agreement with the WHO’s definition [2] where it is defined as physical, sexual or psychological, or emotional violence, or threats of physical or sexual violence that are inflicted on a pregnant woman by a family member, i.e. an intimate male partner, marital/cohabiting partner, parents, siblings, or a person very well known to the family, or a significant other, (i.e. former partner) when such violence often takes place in the home. Intimate partner violence (IPV) is included in the definition of DV. According to Swedish law, interpersonal violence is a criminal act [3] and for a child to grow up in a DV situation not only jeopardizes the health and the development of the child, but it is also a crime against the child [4]. According to Swedish law, a child who witnesses DV is a victim of a crime (ibid). Violence, perpetrated on the pregnant woman and directly or indirectly upon the unborn baby, can lead to serious consequences for their health [5–7]. The mothers-to-be’s health and wellbeing also reflects on the offspring’s health in the womb as well as after birth [8]. It is almost 1.5 times more likely to have a preterm baby and/or a low-birth-weight baby when exposed to DV during pregnancy [5].

In a meta-analysis of 55 independent studies, the most robust predictor for DV among pregnant women was a history of violence [9]. Several other risk factors for DV among pregnant women were identified such as; to be single, have a low standard of education as well as a low socioeconomic status and having an unintended pregnancy [9]. Another systematic review and meta-analysis disclosed that high levels of anxiety, symptoms of perinatal depression as well as posttraumatic stress disorder (PTSD) were significantly associated with the experience of DV during a woman’s lifespan, including while being pregnant [10]. A recently published systematic review including 43 selected studies, suggests that women who have experienced lifetime abuse have a significantly increased risk for depression during the pre-natal and postpartum period when compared to women without a history of abuse [11].

The prevalence of violence against pregnant women is lower, 13.3 %, in the developed countries compared to 27.7 % in the less developed countries [9]. The meta-analysis of 92 independent studies involving 23 countries (Sweden included) showed the average prevalence of DV during pregnancy to be 19.8 %. (ibid). However, cultural dissimilarities can make it problematic to compare prevalence rates across different countries as can variances in the methodology and definitions used. In our former studies (the first and the second parts of this project), the prevalence of DV during early pregnancy was shown to be 1 % in early [12] and 2 % in late pregnancy [13]. Another report from six European countries that used a considerable wider definition for the duration of experienced violence among pregnant women showed a prevalence in Sweden of 3.0 % which was the same as in Belgium and in Iceland, whereas in Denmark it was 3.3 %, in Norway 3.7 % and in Estonia 6.5 % [14]. However, the data is not truly comparable as the time point for the recruitment to the studies differs as well as the contexts. The duration for the experienced abuse was defined in a much wider way or included any experienced abuse over the last 12 months and further the perpetrator was not defined. A British longitudinal study reported the prevalence of physical DV to be 1 % during pregnancy compared to 3 % three years postpartum [15]. However, experience of any form of violence was reported to higher extent or 5.1 % (ibid). The postpartum period is not a violence free period for women [15–18]. In a national Swedish survey undertaken for more than a decade ago (based on one single question), focused on mothers with infants up to 1 year old, at least two percent of mothers were physically abused by their intimate partner [18]. International figures for the prevalence of IPV in the postpartum period from developed countries reported lower or much higher figures than those of Sweden; in a national sample of Canadian women, the figure of any abuse was 1 %, responded during 5 to 14 months postpartum [19]. A study from 16 U.S. cities found that physical IPV (solely) was experienced by 3.1 %, emotional abuse by 27 % and coercion-control behaviour by 41.0 % during a 12 month period postpartum [20]. Among Australian women 17 % experienced, physical and/or emotional, abuse by IPV during the first year postpartum [21].

Pregnancy obviously offers no protection against DV, and therefore can only be viewed as a continuum of already existing violence [12, 22], either decreasing [12, 15], or increasing [6, 23] or beginning during pregnancy [24, 25]. Nevertheless, DV is a significant threat against the health of the pregnant woman and her unborn child [5, 10, 26–29]. If violence already exists within the family, it can equally increase [15] as decrease [19, 30] after delivery.

There are no earlier published national population-based *longitudinal* cohort studies conducted among pregnant and newly delivered women that reveal both the prevalence and incidence of DV, as well as possible risk factors for DV during pregnancy and up to 1–1.5 years postpartum.

The *aim* of this study was to explore prevalence and incidence of domestic violence during pregnancy and 1 to 1.5 years postpartum as well as to explore the history of violence among new mothers in the southwestern region of Sweden. In addition, the aim was to explore the association between domestic violence postpartum and possible risk factors.

## Methods

### Design and setting

The present cohort study has a longitudinal design and represents the third report in the project entitled *“Pregnant women and new mother’s health and life experience”.* The data collection was performed in the southwest area of Sweden*.* The recruitment as well as the setting and the study participants are described in detail elsewhere [12]. The catchment area is characterized by multicultural diversity and the population includes registered women at an ANC from both a University City and an industrial city as well as the smaller surrounding municipalities.

### The characteristics of participants

The inclusion criteria were women ≥ 18 years of age, registered at Antenatal Care (ANC) when pregnant and who could understand and write Swedish or English. Nearly 80 % of the participants had Sweden as their country of origin and the remaining women were born in 93 different other countries [12].

### The process of recruitment

Power calculations showed that at least 2000 participants were needed for statistical calculations in order to achieve with 98 % certainty at least 2.5 % prevalence of DV.

**Fig. 1:**
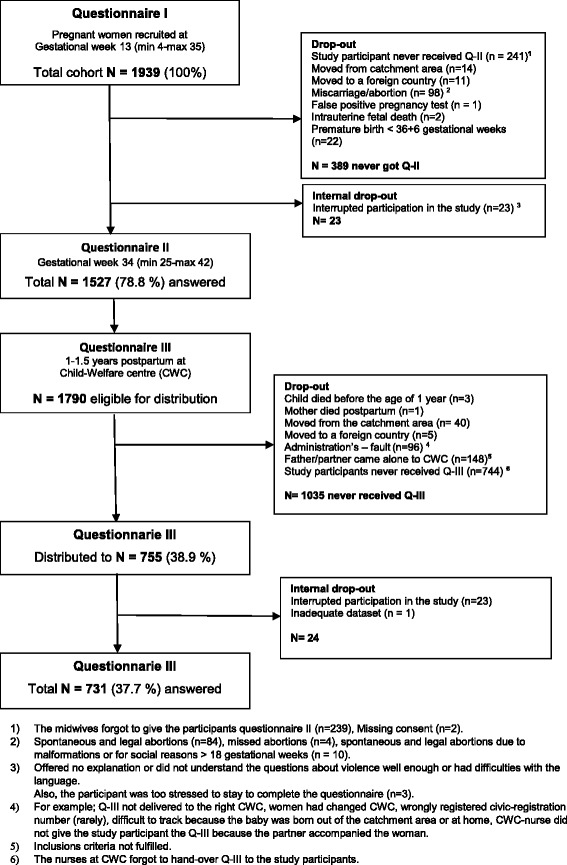
Flowchart over distributed and received answers in Questionnaires I-III

Between March 2012 and September 2013 both primipara and multipara women were recruited while in *early pregnancy,* i.e. gestational week 13 (mean 12.8 weeks, SD 5.11) and were requested to answer Questionnaire I (Q-I) in a private place at the ANC (*N* = 1939). The second Questionnaire II (Q-II) was completed during *late pregnancy* at gestation week 34 (mean 33.9 weeks, SD 2.2). The response rate for Q-II was 78.8 % (*N* = 1527). As a final point, 732 mothers who visited 65 different Child-Welfare-Centers (CWC) with their child/children when they were 1 years old completed the third and the last Questionnaire III (Q-III) at the end of April 2015. One dataset was incomplete which resulted in a total of 731 completed answers of Q-III. Q-III was also completed in a private place (the facilities for privacy varied). If the Child-Welfare nurse missed the opportunity to hand over the Q-III at the 1st year’s routine visit, or if only the father/partner was present, the opportunity was given for the mother to hand over the questionnaire at the next visit, normally at 18 months after the child was born (Fig. 1). If the mother came alone with the child or the intimate partner (irrespective of gender) accompanied the mother to the CWC, the Child-Welfare nurse was permitted to hand over the Q-III to the woman in order for her to complete it at the CWC before going home. According to the WHO ethical and safety recommendations for research on DV against women, it would be strictly forbidden to complete the Q-III at home [31].

### Questionnaires

All the self-reported questionnaires (Q-I, Q-II and Q-III) were completed in as private a place as possible at the ANC’s and CWC’s. Once the participants had completed Q-I, they were familiar with the questions related to any experience of domestic violence. The number of questions were reduced from 122 in Q-I to 93 in Q-II and then increased to 96 in the Q-III. Background questions and the Sense of Coherence scale (SOC-13) [32] were excluded in Q-II and Q-III and questions about breast-feeding were added to Q-III (questions about breastfeeding are not analyzed in the current study). The main instrument the NorVold Abuse Questionnaire (NorAQ) was used, this questionnaire has shown good reliability, validity and specificity regarding the abuse variables [33] and is thoroughly described in our previous study [12]. Questions about all types of violence; psychological, physical and sexual abuse are included in the study as well as the severity of the violence. One additional question, modified [16] from the Abuse Assessment Screen (AAS) [34] was used to investigate current abuse during pregnancy; *Have you been exposed to abuse during current pregnancy?* In order to investigate any emotional, physical and sexual abuse (yes/no, if yes by whom) was added to the questionnaires (ibid). The Edinburgh Postnatal Depression Scale (EPDS) [35], also used during pregnancy (EDS) [36] as well as the Alcohol Use Disorders Identification Test (AUDIT) [37] were added to the questionnaires and described in detail elsewhere [12].

### Definitions

According to Swahnberg et al’s [33] definitions for severity of abuse, which classifies abuse as mild, moderate or severe and the type of abuse was used in the current study. *A history of violence* is defined as a lifetime experience of emotional, physical or sexual abuse occurring during childhood (<18 years), adulthood (≥18 years) or both, regardless of the level of abuse or the perpetrator’s identity, in accordance with the operationalization of the questions in the NorAQ (ibid).

### Classification of the variables

In this study we have used the Same classification of variables as used in our earlier study [12] which were; *Age,* classified and dichotomized as 18–34 and ≥ 35 years, *Language,* as a foreign language spoken at home or Swedish (solely), *Educational status,* as a low educational status, i.e. basic education versus a high educational status such as high school or university. *Cohabiting status* was classified as being single/living apart, or as a common law spouse/married. *Employment status* was dichotomized as being employed (including parental leave and studying) or unemployed (including long-term illness). *Financial distress* was dichotomized as “no” (no problem) or “yes” (serious financial distress). Maternal characteristics concerning body mass index (BMI) were calculated from maternal weight postpartum and height and classified according to WHO’s definition [38] as underweight (<18.5), normal weight (18.50–24.99), overweight (≥25–29.99), and obese (≥30) and dichotomized as under-/normal weight or overweight/obese. *Smoking/using wet tobacco* was dichotomized as “yes” versus “no”, “yes” (if the woman was a daily smoker or wet-tobacco user at some point during pregnancy) and “no” (never smoked/ or used wet-tobacco or stopped before pregnancy). *Alcohol consumption* was dichotomized as “yes” (at least once a month) or “no”. *Unintended pregnancy* was dichotomized as “yes” or “no”. *Abortion/miscarriage* was classified and dichotomized to “no” or both “miscarriage/abortion”. *Self-reported health* was dichotomized as poor health versus rather good health. *Sleep*, was dichotomized as lack of sleep (during the last year, to such an extent that they had problems coping with their daily life), versus adequate sleep*.*

### Statistical methods

Descriptive statistics were utilized to show the prevalence and severity of a lifetime experience of any type and level of abuse. Cochran’s *Q-*test was used to determine whether the proportion of participants who had reported history of abuse were statistically significant between answers received at the three time points at Q-I to Q-III. OR and 95 % CI were calculated for the crude associations between possible risk factors and ‘DV postpartum’, with ‘DV postpartum’ as a dependent variable for bivariate logistic regression. For the purpose of bivariate logistic regression, a variable for depression was computed based on EPDS scores, i.e. symptoms of depression postpartum, whereby an optimal cut-off of ≥ 13 was chosen as representing the presence of symptoms of depression [36]. The EPDS score was computed only for those responding to all ten questions (missing = 66). In order to analyze the association between the SOC score and exposure to ‘DV postpartum’, the SOC-scale was dichotomized utilizing the first quartile of the distribution as a cut-off value (SOC ≤ 64 and SOC >64) [39]. The SOC score was only computed for those responding to all thirteen items (missing = 47). Multiple logistic regression was performed in order to evaluate the influence of variables that were significant in the bivariate logistic regression except for “Lack of sleep and Age” with ‘DV postpartum’ as a dependent variable; the multiple logistic regression analyses were thus step-wise adjusted (forward selection) for; Single/living apart, EPDS ≥ 13, Low SOC-score, as well as Lack of sleep (not significant), and Age (not significant). Statistical significance was accepted at *p* < 0.05. Statistical analyses were performed using the Statistical Package for Social Sciences (SPSS) version 22.0 for Windows.

## Results

The response rate for those women who actually received the Q-III (*n* = 755) at the CWC was almost 97 %. Internal dropout was 23 women who interrupted their participation in the study postpartum and one dataset was incomplete. Of the total cohort (*n* = 1939) of women who were recruited in *early pregnancy* and who answered Q-III there were 1790 women eligible to get Q-III, but 41.6 % (*n* = 744), never received the Q-III due to the nurses at the CWC forgetting to give the questionnaire to the participants (Fig. 1). Drop-out analyses showed that those who did not complete the study and did not answer the third and last questionnaire Q-III had a statistically significant higher education and were to a lesser extent unemployed (Table 1).

**Table 1:** Dropout figures and women who remained throughout the study and answered Q-III (*N* = 1939)

Characteristics	Total n (%) 1939 (100)	Drop-out n (%) 1208 (62.3)	Answered^a^ Q-III n (%) 731 (731.7)	*P*-value χ2
Age				
18–25	342 (17.9)	201(16.8)	141 (19.6)	NS
26–34	1218 (63.7)	781 (65.4)	437 (60.8)	
≥ 35	353 (18.5)	212 (17.8)	141 (19.6)	
Parity				
Primiparae	819 (45.8)	518 (46.6)	301 (44.5)	NS
Multiparae	969 (54.2)	518 (46.6)	376 (55.5)	
Country of origin				
Sweden	1549 (80.1)	967 (80.2)	582 (79.8)	NS
Nordic countries	47 (2.4)	33 (2.7)	14 (1.9)	
Other countries	338 (17.5)	205 (17.0)	133 (18.2)	
Cohabiting status				
Common law spouse/married	1794 (92.5)	1108 (94.6)	662 (93.6)	NS
Single/Living apart	99 (5.1)	63 (5.4)	45 (6.4)	
Educational status				
≤ High school	642 (33.2)	369 (30.6)	273 (37.4)	0.002
University	1293 (66.8)	837 (69.4)	456 (62.6)	
Employment status				
Employed	1827 (94.4)	1150 (95.2)	677 (93.0)	0.042
Unemployed	109 (5.6)	58 (4.8)	51 (7.0)	
Smoking/Snuffing				
No	1523 (78.5)	957 (81.9)	566 (79.7)	NS
Yes	355 (18.3)	211 (18.1)	144 (20.3)	
Use of alcohol				
No	776 (40.0)	538 (46.4)	345 (48.7)	NS
Yes	1102 (56.8)	622 (53.6)	364 (51.3)	
Unintended pregnancy				
No	1576 (82.4)	980 (82.1)	596 (82.9)	NS
Yes	336 (17.6)	213 (17.9)	123 817.1)	
Abortion/miscarriage				
No	1760 (93.5)	1101 (93.7)	659 (93.1)	NS
Yes	123 (6.5)	74 (6.3)	49 (6.9)	

Statistical significance accepted at *p* < 0.05, two-tailed

^a^Women’s status in early pregnancy (Q-I). Missing answers are between 3–151

Table 2 provides a summary of the type and severity of lifetime abuse. History of violence was reported by 33.5 % (*n* = 241) 1 to 1.5 years postpartum. Self-reported experience of any abuse during the past year was 4.2 % (*n* = 31). A Cochran’s *Q* test determined that there was a statistically significant difference in the proportion of women who reported lifetime experience of abuse over time, *p <* 0.0005. Also there was a statistically significant difference in the proportion of women who reported lifetime physical abuse over time, *p <* 0.0005. There were no statistically significant differences in the proportion of women who reported lifetime experience of emotional and sexual abuse.

**Table 2:** Type and severity of history of violence: in Questionnaire I-III

Type and severity of abuse		Questionnaire I Early pregnancy	Questionnaire II Late pregnancy	Questionnaire III 1.5 year pp
	*Missing*	*n* (%)	*n* (%)	*n* (%)
		1928 (100) 11^a^	1497 (100) 30^a^	720 (100) 12^a^
Lifetime experience of abuse^b^		761 (39.5)	562 (36.8)	241 (33.5)
Any abuse during the past year		84 (4.3)	38 (2.5)	31 (4.2)
Lifetime of emotional abuse		374 (19.5)	257 (16.8)	113 (16.0)
*Mild*		307 (16.1)	221 (14.5)	100 (14,1)
*Moderate*		187 (9.8)	123 (8.1)	63 (8.9)
*Severe*		203 (10.6)	135 (8.8)	67 (9.4)
Any emotional abuse during the past year		61 (3.1)	28 (1.8)	23 (3.1)
Lifetime of physical abuse		561 (29.3)	417 (27.3)	177 (24.8)
*Mild*		529 (28.0)	399 (26.1)	170 (24.3)
*Moderate*		203 (10.7)	171 (11.2)	78 (11.0)
*Severe*		127 (6.7)	89 (5.8)	49 (7.0)
Any physical abuse during the past year		36 (1.9)	13 (0.9)	10 (5.3)
Lifetime of sexual abuse		302 (15.7)	218 (14.3)	99 (14.0)
*Mild* ^*c*^		49 (2.6)	37 (2.4)	22 (3.2)
*Mild* ^*d*^		208 (11.0)	169 (11.1)	71 (9.7)
*Moderate*		212 (10.9)	166 (10.9)	73 (10.0)
*Severe*		144 (7.4)	94 (6.2)	38 (5.4)
Any sexual abuse during past year		2 (0.1)	5 (0.3)	3 (2.8)

^a^Not answered the questions about violence

^b^Any type of self-reported abuse during lifetime irrespective perpetrator

^c^Emotional or sexual humiliation

^d^No genital contact

### Prevalence and incidence of DV during pregnancy up to 1–1.5 years postpartum

When all three questionnaires (Q-I to Q-III) were answered, the prevalence of DV during pregnancy (solely) irrespective of type or severity was reported by 2.5 % of the participants (*n* = 40). One to 1.5 years postpartum the prevalence of DV, in the whole cohort, had increased to 3.3 % (*n* = 23), but also the cohort had decreased in number since their recruitment in early pregnancy (Table 3). A Cochran’s Q test determined that there was no statistically significant difference of women who reported DV at the three time points for Q-I to Q-III. The incidence of DV during pregnancy between early pregnancy (Q-I) and late pregnancy (Q-II) was 11 cases as well as between late pregnancy (Q-II) and delivery (Q-III). A total of 22 new cases of self-reported DV during pregnancy gave an incidence of 14 new cases per 1000 women. In the postpartum period, up to 1–1.5 years postpartum, there were 12 new cases of reported DV, which gives an incidence of 17.2 new cases per 1000 women postpartum (the incidence rate is exclusively presented in the text).

**Table 3:** Prevalence of DV during pregnancy and 1–1.5 year postpartum^a^ (*N* = 1939)

Characteristics	Prevalence of DV early pregnancy	Prevalence of DV late pregnancy^b^	Prevalence of DV during pregnancy^c^	Prevalence of DV 1.5 years pp
	*n* (%)	*n* (%)	*n* (%)	*n* (%)
In the analysis	1928 (99.4)	1467 (75.7)	1573 (81.1)	697 (35.9)
*Missing* ^*d*^	11 (0.6)	472 (24.3)	336 (18.9)	35 (4.8 )
Emotional abuse	15 (0.8)	24 (1.6)	36 (2.3)	18 (2.6)
Physical abuse	7 (0.4)	11 (0.7)	13 (0.8)	8 (1.2)
Sexual abuse	2 (0.1)	2 (0.1)	2 (0.1)	2 (0.3)
Total of any type of abuse	18 (1.0)	29 (2.0)	40 (2.5)	23 (3.3)

^a^Some women may report more than one type of violence

^b^Self-reported at least once in Q-I, Q-II or both questionnaires

^c^Self-reported at least once in Q-I, Q-II, Q-III or in all questionnaires

^d^Excluded in the analysis, because the questions about violence or the whole questionnaire were not answered

### Association between possible risk factors and exposure to DV postpartum

The single strongest risk factor for DV reported at 1–1.5 years postpartum was *a history of violence* whereby all of the women (*n* = 23) who had revealed exposure to DV postpartum had also reported a history of violence (*p* < 0.001). Women who were *Single/living apart* were almost 13 times more likely to report exposure to DV postpartum (*p* < 0.001). In addition, women having an EPDS-score of ≥ 13 indicating the presence of several symptoms of depression were 6.2 times more likely to be exposed to DV postpartum (*p* < 0.001). Of those 9.9 % (*n* = 66) who had high scores on EPDS ≥ 13 postpartum, 62 % (*n* = 41) had solely reported high scores in Q-III. Finally, women having a low score on the SOC-scale (in early pregnancy), indicating an inability to use their own resources to maintain and improve their health in stressful situations were 5.2 times more likely to be exposed to DV postpartum (*p* < 0.001) (Table 4).

**Table 4:** Association between possible risk factors and DV 1–1.5 years postpartum (*N* = 731).^a^

		DV		
		1–1.5 year postpartum		*P*-value
Independent variable	*n* (%)	*n* (%)	OR 95 % CI^b^	(two-tailed)
History of violence^c^	241 (33.5)	23 (9.5)	-	<0.001
Age ≥ 35	141 (19.6)	3 (2.1)	0.6 (0.2–2.1)	NS
Multiparae	376 (55.5)	11 (2.9)	0.8 (0.3–1.9)	NS
Low educational status	273 (37.4)	10 (3.7)	0.8 (0.3–1.8)	NS
Unemployed	51 (7.0)	4 (7.8)	2.9 (1.0–9.0)	NS
Foreign language	183 (25.2)	9 (4.9)	2.0 (0.8–4.6)	NS
Single/living apart	36 (4.9)	8 (22.2)	12.8 (5.0–32.7)	<0.001
Financial distress	357 (49.0)	15 (4.2)	2.0 (0.8–4.7)	NS
Alcohol consumption^d^	481 (66.8)	13 (2.7)	0.7 (0.3–1.7)	NS
Smoking/using wet tobacco	142 (19.6)	5 (3.5)	1.1 (0.4–3.1)	NS
Overweight/obese	238 (32.6)	5 (2.1)	0.5 (0.2–1.4)	NS
Unintended pregnancy	123 (17.1)	3 (2.4)	0.7 (0.2–2.5)	NS
Miscarriage/abortion	49 (6.9)	1 (2.0)	0.6 (0.1–4.6)	NS
Self-reported poor health	62 (8.8)	3 (4.8)	1.8 (0.5–6.2)	NS
Lack of sleep	79 (11.2)	5 (6.3)	2.4 (0.9–6.7)	NS
EPDS ≥ 13	66 (9.9)	8 (12.1)	6.2 (2.5–15.6)	<0.001
SOC Low score^e^	190 (27.8)	15 (7.9)	5.2 (2.2–12.5)	<0.001

^a^Missing system = 12

^b^Method used: bivariate logistic regression showing unadjusted univariable odds ratios and confidence intervals

^c^All (*n* = 23) reported history of violence and therefore OR with 95 % CI not showed

^d^At least once a month

^e^SOC-score measured in early pregnancy

The following variables were checked in a multiple regression analysis; Single/living apart, EPDS ≥ 13, Low SOC-score, Lack of sleep, and Age (excluded from the model). Model 1: The strongest predictor was single/living apart as an independent variable according to the *p*-value in Table 4 remaining significant (*p* < 0.001) and had a 12.9 times higher risk of being associated with DV postpartum. Model II: The second strongest predictor was EPDS ≥ 13 (*p* < 0.03), included in the model and had had a 3.5 times higher risk of being associated with DV postpartum. Model III: The third strongest predictor, Low score SOC (*p* < 0.03) was added to the model, and had a 3.0 times higher risk of being associated with DV postpartum. In model II and III the effect remains by both EPDS and low SOC-scores. In model IV the variable Lack of sleep was added to the model but remained non-significant (Table 5).

**Table 5:** Association between possible risk factors and exposure to DV 1–1.5 years postpartum (*N* = 23)

Variables	Model I OR (95 % CI)	Model II OR (95 % CI)	Model III OR (95 % CI)	Model IV OR (95 % CI)
Single/living apart ^a^	12.8 (5.0–32.7)	12.1 (4.4–33.5)	12.2 (4.4–34.2)	12.9 (4.5–37.1)
EPDS ≥ 13 ^b^		4.5 (1.6–12.1)	3.3 (1.2–9.2)	3.5 (1.2–10.4)
Low score SOC ^c^			2.9 (1.1–7.7)	3.0 (1.1–8.3)
Lack of sleep ^d^				0.7 (0.2–2.5)

^a^Single/living apart versus cohabiting (reference category)

^b^EPDS ≥13, indicating having a risk of depression versus not ≤ 13 (reference category)

^c^Low score SOC indicating inability to use their own resources to maintain and improve their health in stressful situations versus medium-high score (reference category). Questions answered in early pregnancy

^d^Lack of sleep versus adequate sleep (reference category)

### Women separated from their partners during pregnancy or postpartum

Of 731 women who answered both QI and QIII, thirteen had separated from their partner and nine had become a common law spouse/married. Further, of those women who revealed that they were exposed to DV postpartum (*n* = 23), four of them had separated from their partner postpartum. Additionally, one woman had separated during pregnancy (only presented in the text).

## Discussion

This is the first Swedish longitudinal study with the aim to explore both the prevalence and incidence of DV during pregnancy and up to 1–1.5 years postpartum as well as to explore possible risk factors. The prevalence of DV during pregnancy (solely) irrespective of type or severity was revealed by 2.5 % of the participants (reported in gestation weeks: 13, 34 and until directly after delivery). In actual figures this means that statistically at least 225 pregnant women in the catchment area are exposed to DV during pregnancy annually (calculated on 9000 deliveries) and that the prevalence has increased since our last two reports [12, 13]. This indicates that the violence is not only a continuum of violence [12, 22], but increases as the pregnancy advances which is supported by earlier research [6, 23]. However, the figures agree with those of earlier research carried out in northern Europe, such as that 5.1 % of emotional and physical cruelty from a partner during pregnancy was reported from a longitudinal study from England [15]. In Norway, the prevalence of violence during pregnancy was reported to be 5 % [40] and in a cohort of primipara Danish women the prevalence of violence-exposed pregnant women was 2.5 % [24]. According to the present study’s results the prevalence of DV during pregnancy in Sweden is as common as gestational diabetes (In Sweden/Scania prevalence 1.1 and 2.7 % respectively) and almost as common as preeclampsia (In Sweden/Scania prevalence 2.8 and 2.7 respectively) [41]. Awareness of this fact is very important for working midwives in clinical practise as well as other health care providers. Therefore, to identify, support and guide the women exposed to violence to the right authority and thereby prevent any complications that violence can cause to both the woman and the unborn or new-born baby.

The present study also revealed that the prevalence of DV increased from 2.5 % during pregnancy to 3.3 % up to 1–1.5 years postpartum. However, the study cohort had decreased considerably since recruitment in early pregnancy, but the figures are small and there was no statistically significant difference among women who reported DV during pregnancy compared to postpartum. Hypothetically, it is possible that the prevalence of violence has now, 1–1.5 years postpartum, returned to the earlier levels experienced by non-pregnant women before pregnancy. In addition, in the present study there is an indication that the prevalence of DV 1–1.5 years postpartum might be underestimated because 4.2 % of the newly delivered women revealed experience of abuse in the past year. It is a possibility that a lost to follow-up participants might have been abused and therefore the prevalence and the incidence is reported lower. That violence increases during the postpartum period is supported by earlier research from England [15]. An earlier report from Sweden revealed the prevalence of a 2 % exposure to violence at 1 year postpartum [18] as well as a retrospective study with a national sample from Canada revealing decreased prevalence of DV during pregnancy of 1.4 to 1 % postpartum [19]. Cultural differences as well as differences in the methodology used can make it difficult to compare prevalence rates across countries and different contexts. Sweden, is known internationally for its democracy and gender equality [42], which may have an impact on the huge differences in prevalence of violence compared to Australia [21] and U.S. [20]. However, it cannot be the only explanation. For example, when comparing the US with Sweden there are major differences between the two societies, for example the general acceptance of the possession of weapons. Therefore, it is important to undertake research in domestic violence in different contexts, in each different country. Nevertheless, awareness by the personnel at the CWC about the possible existence of DV is crucial for both the mother’s and the child’s health and welfare.

The current study revealed an incident rate of 14 new cases per 1000 women of self-reported DV during pregnancy as well as an incident rate of additional 17.2 new cases per 1000 women up to 1–1.5 years postpartum. In actual figures, this means that at least 126 new cases during pregnancy respectively 155 women postpartum will be exposed to DV annually in the catchment area. In addition, this indicates that the violence-exposed women becomes aware of the violence and admit that they are violence-exposed, dependent on the fact that they are repeatedly asked about violence over time, which is supported by a Cochrane review [43]. If the health-care providers ask sensitive questions early in the pregnancy and repeat them later in pregnancy as well as postpartum, the violence-exposed women may become more aware of their difficult situation and the possible stigma surrounding the subject will decrease, and the victims of violence may ask for support in their difficult situation. It would be helpful if the philosophy of maternity care was women-centered thereby underpinning the one-to one- relationship with the woman as well as the focus on the women’s needs, expectations and aspirations [44].

One of three women who answered Q-III (the last questionnaire) self-reported a lifetime experience of violence. This is supported by a WHO report [45] related to the global prevalence of violence against women. However, in the current study the cohort has decreased over time as well as the reported prevalence of lifetime experience of violence, which was almost 40 % in early pregnancy compared to 36.8 % in late pregnancy and finally 33.5 % postpartum. Nevertheless, it is disturbing to know that three to four out of ten women, the midwives meet in early pregnancy, may have unprocessed experience of abuse, which could influence their health during pregnancy. This should be taken into account when the midwife discloses a history of violence.

Not surprisingly, a history of violence was the single strongest risk factor for DV during pregnancy, which is supported by the literature [9]. Therefore, it is important to ask the pregnant women at their first visit to the ANC about their lifetime experience of violence as a part of the anamnesis. It is almost thirteen times more likely for the woman to be single/living apart if exposed to DV during the postpartum period, which is supported by earlier research [9].

Assuming that the cultural context of Swedish society is internationally recognized as being gender-equal and that the women are generally well educated and independent. As this is the case, some women choose, not to cohabit, or be married despite their being pregnant, for example if their relationship is rather new. However, it is extremely important for health care providers to be aware that single women/living apart should be seen as a risk group for abuse. Already this group is vulnerable, as a single parent to be, and therefore individually prepared support should be offered.

In the current study at least five out of 13 women who had ended their partnership at the time they answered the last questionnaire 1–1.5 years postpartum also had experience of abuse during the study period. Therefore, the DV could have been a reason, for those women, for ending their partnership. Four of the five ended their relationship postpartum. This is in line with our previous results where it was shown that women exposed to violence during pregnancy were not willing to leave the perpetrator of the violence during pregnancy, as they believe that it is best for the baby that they stay in the relationship until postpartum [6]. The result indicating that women who were single/living apart were 13 times at higher risk for abuse than a cohabiting/married women may have several hypothetical explanations.

Still fifteen of 23 women who were exposed to DV postpartum remain in their violent relationship. As earlier research has shown, those women who are living with the perpetrator during their pregnancy are very isolated and lack social support as well as being unwilling to leave the perpetrator during the pregnancy for the sake of the unborn baby that they believe they are protecting [6]. In addition, self-blame and shame are parts of this complex social behavior (ibid). In addition, in the current study, the results revealed that women who had several symptoms of depression were 3.5 times more likely to be exposed to DV postpartum and this association is supported by a systematic review [9]. As well, another systematic review indicates that there is an association between maternal lifetime abuse and depressive symptoms during the perinatal period [11]. Depression during the perinatal period is of special importance due to the negative effects on the mother’s health as well as the risk of a negative health outcome for the child.

### Strength and weakness in the study

This is a longitudinally designed study based on prospectively collected data, which allows comparison of pregnant and newly delivered women who are exposed to violence with those who are not throughout the same time-period, which, is considered as a strength to the study as it offers the possibility to explore both the prevalence and incidence of violence. In addition, using validated instruments in the questionnaires [32–35, 37, 46] where the main instrument has been previously used in a multi-country study, [47] and validated within a Swedish population, [33] is also considered to be a strength. However, there were 1790 study participants eligible to complete the last Q-III, but no less than 1035 women never received the questionnaire. The main reason being that the nurses at the CWC’s forgot to hand-over the Q-III to the study participants (*n* = 744) or it was their partner (*n* = 148) who took the child to a CWC instead of the mother or both parents (Fig. 1). This may reflect how strained the CW-nurses working situation was and highlights the result of many staff changes due to vacation and sick leave. The cohort was considerably reduced from Q-I to Q-III and Q-III was distributed to a cohort of 755 women or about 39 % of the original cohort. However, of those women who received the Q-III, there were only about 3 % who did not answer it which can be regarded as very good response rate. Due to the cohort being reduced from initially 1939 participants who answered Q-I to 731 participants who answered the Q-III we must allow for the fact that the prevalence and incidence of DV postpartum may be underestimated.

## Conclusion

Pregnancy as well as the postpartum period up to 1–1.5 years are no free zones for domestic violence. Domestic violence increases as the pregnancy develops as well as in the postpartum period. A history of violence and being single/living apart are the strongest risk factors for domestic violence during pregnancy as well as postpartum. In addition, having several symptoms of depression has an association to domestic violence both during pregnancy and postpartum. Collaboration between health care providers at Antenatal-care and Child-Welfare Centres is essential. In order to improve maternal and child health there is a real need to address this vulnerable group of women both at the ANCs and at CWCs.

### Implications

The health care providers play a crucial role for the survival of mothers and children faced with domestic violence not only to prevent the progression of the violence but also to empower the survivors and give them individually planned support. In the postpartum period up to 1–1.5 years, there is a need for special attention to those women who have a history of violence and are single/living apart, as well as those who show several types of depression symptoms.

## Abbreviations

AAS: Abuse Assessment Screen
ANC: Antenatal Care
AUDIT: Alcohol Use Disorders Identification Test
BMI: Body mass index
CI: Confidence intervals
DV: Domestic violence
EDS: Edinburgh Depression Scale
EPDS: Edinburgh Postnatal Depression Scale
NorAQ: NorVold Abuse Questionnaire
OR: Odds ratios
Q-I: Questionnaire I
Q-II: Questionnaire II
Q-III: Questionnaire III
SOC-13: Sense of Coherence Scale-short form
WHO: World Health Organization
