# Erratum to: Increasing prevalence and incidence of domestic violence during the pregnancy and one and a half year postpartum, as well as risk factors: −a longitudinal cohort study in Southern Sweden

**DOI:** 10.1186/s12884-016-1175-6

**Published:** 2016-12-05

**Authors:** Hafrún Finnbogadóttir, Anna-Karin Dykes

**Affiliations:** 1 Department of Care Science, Faculty of Health and Society, Malmoe University, Malmoe, Sweden; 2 Department of Health Sciences, Medical Faculty, Lund University, Lund, Sweden

## Erratum

In the original publication of this article [1], the table heading in the fourth column of Table 1 should have been “Answered^a^ Q-III n (%) 731 (37.7)”, and not “Answered^a^ Q-III n (%) 731 (731.7)”. Please see updated table below; this has also been updated in the original article accordingly.

**Table 1:** Dropout figures and women who remained throughout the study and answered Q-III (N = 1939)

Characteristics	Total n (%) 1939 (100)	Drop-out n (%) 1208 (62.3)	Answered^a^ Q-III n (%) 731 (37.7)	*P-*value *χ* ^2^
Age				NS
18–25	342 (17.9)	201(16.8)	141 (19.6)	
26–34	1218 (63.7)	781 (65.4)	437 (60.8)	
≥ 35	353 (18.5)	212 (17.8)	141 (19.6)	
Parity				NS
Primiparae	819 (45.8)	518 (46.6)	301 (44.5)	
Multiparae	969 (54.2)	593 (53.4)	376 (55.5)	
Country of origin				NS
Sweden	1549 (80.1)	967 (80.2)	582 (79.8)	
Nordic countries	47 (2.4)	33 (2.7)	14 (1.9)	
Other countries	338 (17.5)	205 (17.0)	133 (18.2)	
Cohabiting status				NS
Common law spouse/married	1794 (92.5)	1108 (94.6)	662 (93.6)	
Single/Living apart	99 (5.1)	63 (5.4)	45 (6.4)	
Educational status				0.002
≤ High school	642 (33.2)	369 (30.6)	273 (37.4)	
University	1293 (66.8)	837 (69.4)	456 (62.6)	
Employment status				0.042
Employed	1827 (94.4)	1150 (95.2)	677 (93.0)	
Unemployed	109 (5.6)	58 (4.8)	51 (7.0)	
Smoking/Snuffing				NS
No	1523 (78.5)	957 (81.9)	566 (79.7)	
Yes	355 (18.3)	211 (18.1)	144 (20.3)	
Use of alcohol				NS
No	776 (40.0)	538 (46.4)	345 (48.7)	
Yes	1102 (56.8)	622 (53.6)	364 (51.3)	
Unintended pregnancy				NS
No	1576 (82.4)	980 (82.1)	596 (82.9)	
Yes	336 (17.6)	213 (17.9)	123 817.1)	
Abortion/miscarriage				NS
No	1760 (93.5)	1101 (93.7)	659 (93.1)	
Yes	123 (6.5)	74 (6.3)	49 (6.9)	

Statistical significance accepted at *p* < 0.05, two-tailed

Missing answers are between 3–151

^a^Women’s status in early pregnancy (Q-I)
